# Expert-based classification on the technical difficulty of single-port urological robotic procedures

**DOI:** 10.1007/s11701-026-04124-8

**Published:** 2026-10-31

**Authors:** Franck Bruyère, Nuno Grilo, Francesco Soria, Marco Oderda, Christopher Anderson, Simone Crivellaro, Ilaria Lucca, Paolo Gontero

**Affiliations:** 1 Department of Urology, Centre Hospitalier Universitaire Vaudois (CHUV), Rue du Bugnon 46, Lausanne, 1011 Switzerland; 2 Department of Urology, School of Medicine, University of Torino, Turin, Italy; 3 Department of Urology, Cromwell Hospital, London, UK; 4 Department of Urology, University of Illinois at Chicago, Chicago, IL USA

**Keywords:** Single-port robotic surgery, da Vinci SP, Urological surgery, Learning curve, Difficulty classification, Expert-based classification

## Abstract

Single-port (SP) robotic surgery represents the latest paradigm shift in minimally invasive urology. Despite its progressive adoption worldwide, no structured classification of procedural difficulty exists for SP urological procedures. We aimed to develop an expert-based scoring system to grade the technical difficulty of urological procedures performed with the da Vinci SP platform. A questionnaire-based expert-consensus study was conducted during an international meeting on SP robotic surgery (Turin, Italy, February 2026). Consensus here refers to the descriptive convergence of independent, single-round ratings rather than a formal iterative (e.g., Delphi) consensus process. Eligible experts had independent or assisted SP robotic experience. Using a structured questionnaire, they rated each procedure on three dimensions — technical difficulty, operative risk and sustained attention required — on a 7-point Likert scale. A composite difficulty score was obtained by summing the three median values. Experts also estimated the number of cases required to reach proficiency and rated the role of prior multi-port robotic experience. Nine procedures were classified. Composite scores ranged from 8 (pyeloplasty, “slightly difficult”) to 16 (radical cystectomy with intracorporeal diversion, “very difficult”). Estimated proficiency thresholds ranged from 10 to 33 cases. All experts considered prior multi-port robotic experience necessary before SP adoption. Early challenges were dominated by prolonged operative time, limited exposure and reduced ergonomics rather than instrument collision or spatial disorientation. We propose the first structured expert-based difficulty classification for SP robotic urological surgery. The procedural hierarchy mirrors historical laparoscopic and multi-port robotic classifications, supporting the concept that intrinsic complexity transcends the platform. This framework may inform training curricula, case selection during adoption and safe dissemination of SP robotic surgery.

## Introduction

Over the past three decades, urological surgery has undergone profound technological transformations that have reshaped surgical practice. The transition from open surgery to conventional laparoscopy marked the first major shift towards minimally invasive approaches. This evolution was followed by the widespread adoption of multi-port robotic surgery, which further enhanced dexterity, visualization and reproducibility. More recently, the introduction of single-port (SP) robotic platforms represents a new stage in this progression, redefining surgical ergonomics, instrument triangulation and operative strategy.

Each technological transition introduces not merely a modification of technique but effectively a new procedure, associated with a distinct learning curve. Mastery of novel platforms requires structured training and careful dissemination to minimize intra-operative challenges and postoperative complications. At the same time, each innovation benefits from the cumulative experience acquired during previous transitions. The learning curve of SP robotic surgery is therefore built upon the conceptual and technical foundations established during the eras of laparoscopy and multi-port robotics.

In 2001, Guillonneau et al. proposed a classification system assessing the technical difficulty of laparoscopic urological procedures based on expert consensus [1]. This framework provided valuable guidance for training, case selection and the structured dissemination of laparoscopic techniques across the urological community. The same principle of progressive, difficulty-graded exposure has since been operationalized in contemporary robotic training curricula: for instance, the ERUS structured curriculum for robot-assisted partial nephrectomy sequences procedural steps into five modules ranked from easy to complex, with progression contingent on demonstrated proficiency at each level [2]. To date, however, no equivalent structured classification exists for urological procedures performed with the SP robotic platform, despite its progressive expansion in Europe and worldwide [3, 4].

Given the specific technical constraints of single-port surgery — including instrument crowding, altered triangulation, ergonomic adaptation and reconstructive challenges — there is a clear need for a pragmatic, expert-based classification system to define procedural difficulty. Such a framework would support structured training pathways, optimize patient selection during the adoption phase and contribute to the safe and rational dissemination of SP robotic urological surgery.

In this context, we aimed to develop a consensus-based scoring system to classify the technical difficulty of urological procedures performed with the da Vinci SP robotic platform.

## Materials and methods

### Study design and setting

This study was designed as a questionnaire-based expert-opinion survey, based on independent, single-round ratings rather than a formal Delphi or iterative consensus process; no consensus threshold or feedback round was applied.

Data collection was conducted during a dedicated scientific meeting on SP robotic surgery held in Turin, Italy, in February 2026. The meeting gathered international urological surgeons with established experience in SP robotic procedures. The objective was to develop a pragmatic scoring system to classify the technical difficulty of urological procedures performed with the da Vinci single-port robotic platform.

### Participants

Experts were eligible if they had independently performed or assisted in at least one SP robotic procedure at the time of the meeting; no minimum case-volume threshold was set a priori, and this eligibility criterion is intentionally broad Participation was voluntary and all respondents completed the survey anonymously; individual-level data on SP or multi-port case volume, years of independent robotic practice, or fellowship training could therefore not be linked to responses. A total of 18 international SP robotic surgeons attending the meeting completed the questionnaire. This precludes formal characterization of panel expertise beyond meeting-attendance eligibility, and is addressed as a primary limitation below.Survey structure.

A structured questionnaire was developed prior to the meeting and distributed electronically. For each selected SP urological procedure, participants rated three dimensions: technical difficulty; operative risk, defined as the probability and severity of intraoperative or early postoperative complications directly attributable to the technical execution of the procedure; and sustained attention, defined as the degree of continuous cognitive concentration and vigilance required throughout the procedure, independent of its physical or technical difficulty (e.g., prolonged, precision-demanding dissection with a low margin for error).Each parameter was rated on a 7-point Likert scale (1 = lowest, 7 = highest). Experts were also asked to estimate the number of cases after which a well-trained multi-port robotic surgeon would typically reach independent, complication-free performance of each SP procedure — a subjective “comfort/proficiency” threshold, without further operational definition provided to respondents. A multiple-choice and open-ended question addressed the main technical difficulties encountered during the initial SP learning phase.

### Development of the difficulty score

For each procedure, the median score was calculated separately for technical difficulty, operative risk and sustained attention required. A composite difficulty score was then obtained by summing the three median values; this aggregate score defined the final difficulty level of each procedure. This equal-weighting approach, adapted from the original Guillonneau laparoscopic difficulty classification [1], assumes that the three domains contribute comparably to overall procedural burden. It is a pragmatic simplification rather than a psychometrically validated or empirically weighted model; alternative weighting schemes were not tested. Because the three component medians are reported individually in Table 1, readers may apply an alternative weighting according to their own clinical priorities.

Procedures were subsequently ranked in ascending order of composite score. The resulting classification is presented in Table 1.

### Statistical analysis

Given the expert-based survey design and the ordinal nature of the data, results are presented using medians. Descriptive statistics were used to summarize responses. No inferential statistical comparisons were planned, as the primary objective was classification rather than hypothesis testing.

### Ethical considerations

As this study involved anonymized expert opinion without patient data, formal research-ethics committee approval was not required according to institutional and national regulations. The study was conducted in accordance with the principles of the Declaration of Helsinki.

## Results

A total of *18* completed the questionnaire. Nine urological procedures were classified. Composite difficulty scores ranged from 8 (pyeloplasty, classified as “slightly difficult”) to 16 (radical cystectomy with intracorporeal diversion, “very difficult”). Estimated proficiency thresholds ranged from a median of 10 cases (pyeloplasty) to 33 cases (radical cystectomy). The complete classification is presented in Table 1.

When asked whether prior multi-port experience was necessary before starting SP surgery, none of the 18 experts rated it as unnecessary. Four (22.2%) rated it as “essential”, five (27.8%) as “necessary”, six (33.3%) as “moderately necessary” and three (16.7%) as “slightly necessary” (median between “moderately necessary” and “necessary”). Overall, half of the panel (9/18) considered prior multi-port experience necessary or essential.In response to the question “During your first single-port (SP) robotic cases, which specific difficulties did you mainly encounter?”, most experts emphasized prolonged operative time, a limited surgical field, and reduced ergonomics and range of motion. Instrument collision, adaptation to visualization and spatial orientation were less frequently cited.

**Table 1:** Median scoring of urological procedures according to technical difficulty, operative risk and attention required, with estimated case numbers to proficiency and the resulting composite classification

Procedure	Median cases to proficiency	Technical difficulty	Operative risk	Attention required	Composite score	Classification
Pyeloplasty	10	3	2	3	8	Slightly difficult
Promontofixation	15	3	3	3	9	Fairly difficult
Ureteric reimplantation	15	3	3	3	9	Fairly difficult
Simple nephrectomy	14	3	3	4	10	Fairly difficult
Radical prostatectomy	27	3	3	5	11	Fairly difficult
Simple prostatectomy	17	4	3	4	11	Fairly difficult
Radical nephrectomy	18	4	3	5	12	Difficult
Partial nephrectomy	24	4	4	5	13	Difficult
Radical cystectomy with intracorporeal diversion	33	5	5	6	16	Very difficult

The composite score is the sum of the median technical difficulty, operative risk and attention scores

## Discussion

The present expert-based survey proposes a structured classification of urological procedures performed with the SP robotic platform, integrating technical difficulty, operative risk and sustained-attention requirements. Several findings emerge as particularly relevant.

Our classification demonstrates a progressive increase in composite difficulty scores from reconstructive and ablative low-complexity procedures (pyeloplasty, promontofixation, ureteric reimplantation) to technically demanding oncological procedures such as radical cystectomy. Radical cystectomy was identified as the most complex procedure (composite score 16), followed by partial nephrectomy (13) and radical nephrectomy (12).

This hierarchical distribution mirrors previously described gradients in laparoscopic and multi-port robotic surgery. In 2001, Guillonneau et al. [1] proposed a difficulty-based classification of laparoscopic urological procedures derived from expert consensus, demonstrating a similar escalation from reconstructive procedures to major oncological surgery. Our findings suggest that, despite technological evolution, intrinsic procedural complexity remains structurally comparable across platforms.

Furthermore, contemporary robotic literature consistently identifies radical cystectomy and partial nephrectomy as among the most technically demanding procedures, owing to reconstructive steps, vascular control and margin-critical dissection [5, 6]. The SP platform does not appear to fundamentally alter this hierarchy but rather modifies the technical constraints through altered ergonomics and triangulation.

Median estimated case numbers required to achieve proficiency ranged from 10 cases (pyeloplasty) to 33 cases (radical cystectomy); notably, radical prostatectomy required a median of 27 cases. These figures are substantially lower than historical learning curves reported for conventional laparoscopy and early robotic surgery: early laparoscopic radical prostatectomy series reported learning curves exceeding 50–100 cases [7, 8]; multi-port robotic radical prostatectomy learning curves have been estimated between 20 and 40 cases for functional stabilization, with oncological optimization often requiring more [9, 10]; and partial nephrectomy learning curves in robotic series frequently exceed 30–40 cases [11].

The comparatively shorter proficiency estimates reported by our panel may reflect cumulative expertise acquired during prior multi-port robotic experience, although this relationship was not directly tested in the present study. This observation is compatible with the concept of “technological inheritance” — whereby each innovation may benefit from previously developed cognitive and technical frameworks [11] — but should be regarded as a plausible explanatory hypothesis rather than a confirmed finding.

All participating experts agreed that prior multi-port robotic experience is necessary before initiating SP surgery, reinforcing the concept that SP surgery should not be considered an entry-level robotic platform. Instead, it appears to represent a refinement of established robotic principles, requiring pre-existing mastery of robotic ergonomics, dissection strategy and reconstructive techniques. This view is consistent with survey data among trainees, in which SP exposure remains concentrated in advanced robotic settings [4].

Published data on the SP learning curve remain limited but are emerging and provide valuable context for our findings. Procedure-specific analyses suggest that learning dynamics are influenced both by the platform and by intrinsic procedural complexity. Pellegrino et al. reported that proficiency milestones — defined by declining complication rates — vary considerably between procedures, with simple reconstructive and renal operations achieving earlier competency and more complex procedures such as SP radical prostatectomy requiring substantially more cases (up to approximately 150) to minimize risk metrics, underscoring the procedure-dependent nature of the SP learning curve [12]. Notably, our median expert-estimated threshold for SP radical prostatectomy (27 cases) is markedly lower than the ~ 150-case threshold reported by Pellegrino et al. based on objective complication-rate stabilization. This discrepancy is informative: our estimates reflect experts’ subjective perception of reaching independent, comfortable practice, whereas objectively measured learning curves based on complication or margin rates may extend considerably further. This distinction should temper any direct clinical application of our proficiency figures and reinforces the need for validation against hard outcome data. Consistent with this, CUSUM-based analyses of SP extraperitoneal radical prostatectomy and updated learning-curve reviews indicate that operative-time reduction, rather than complication rates, often marks the earliest indicator of maturation along the SP learning curve [3, 13].

An important methodological contribution of our study is the inclusion of sustained attention as a third independent dimension alongside technical difficulty and operative risk. Certain procedures (e.g., radical prostatectomy and radical nephrectomy) showed relatively moderate technical-difficulty scores but higher attention requirements, reflecting prolonged precision-demanding dissection phases. Radical cystectomy scored highest across all three domains, confirming its global complexity. This multidimensional approach extends beyond traditional binary “easy versus difficult” classifications and may better reflect real operative cognitive load, a parameter increasingly recognized in surgical-performance research.

The classification proposed in Table 1 provides a pragmatic roadmap for case selection during SP adoption, a structured framework for training curricula, a basis for progressive credentialing, and a reference tool for future comparative studies.

Importantly, the consistency of our ranking with historical laparoscopic classifications suggests that procedural complexity is an inherent property of the operation itself, while the robotic platform modifies the technical pathway but not the fundamental hierarchy — a concept consistent with systematic-review evidence that technical skill, particularly for complex surgical maneuvers, transfers across laparoscopic and robotic platforms [14].

Notably, radical and simple prostatectomy obtained an identical composite score (11) despite fundamental differences in surgical objective and clinical stakes. This convergence reflects different score profiles rather than genuinely equivalent difficulty: radical prostatectomy scored higher on sustained attention (median 5 vs. 4), consistent with the cognitive demands of nerve-sparing dissection and oncological margin control, whereas simple prostatectomy scored higher on technical difficulty (median 4 vs. 3), reflecting the challenges of hemostasis and capsule dissection in large, vascular benign glands without the constraint of oncological margins. This illustrates a broader limitation of the composite score: procedures with different clinical risk profiles and different consequences of technical error (e.g., a positive margin in oncological surgery versus a capsular perforation in benign disease) can converge on the same aggregate value. We therefore recommend that readers consult the individual domain scores in Table 1, rather than the composite alone, whenever the clinical stakes of a specific error type are the primary concern.

Several limitations should be acknowledged. First, the procedures included do not represent an exhaustive list of operations feasible with the SP platform; many other procedures may also be safely undertaken with this approach. Second, the study is based on expert opinion rather than objective perioperative outcomes; however, this methodology mirrors previously accepted frameworks in laparoscopic classification systems. Third, participating experts were already experienced robotic surgeons, which may underestimate the learning curve for less experienced adopters. A key limitation of this study is the small size (*n* = 18) and incomplete characterization of the expert panel. Respondents were self-selected attendees of a single international meeting; to preserve full anonymity, individual-level data on SP case volume, years of independent SP practice, and prior multi-port experience were not collected, precluding formal verification of panel expertise and limiting the generalizability of our findings. A structured, reproducible expert-selection process — for example a minimum case-volume threshold combined with a formal multi-round Delphi panel with documented credentials — would strengthen external validity in future iterations of this classification. The composite score itself has not been formally validated against external criteria (e.g., objective operative outcomes), and its equal-weighting assumption may not hold across all procedure types: radical and simple prostatectomy obtained an identical composite score despite markedly different oncological stakes. Estimated proficiency thresholds were based entirely on retrospective, self-reported expert opinion rather than objective perioperative endpoints (complication rates, warm ischemia time, positive margin rates, conversion rates) and are therefore susceptible to recall bias, particularly for procedures learned years earlier or performed infrequently. These figures should be interpreted as indicative expert impressions rather than validated learning-curve estimates. Finally, each procedure was rated as a single, generic category without accounting for technical variables known to materially affect difficulty — such as transperitoneal versus extraperitoneal access, anterior versus posterior tumor location and complexity (e.g., RENAL/PADUA score) for partial nephrectomy, gland size or prior treatment for prostatectomy, or the specific type of urinary diversion (ileal conduit versus orthotopic neobladder) for radical cystectomy. This simplification was necessary to keep the survey feasible within a single meeting-based questionnaire but limits the granularity and direct clinical applicability of the resulting hierarchy. Future work should stratify difficulty ratings by these procedure-specific technical variables.

Future studies should correlate this expert-derived difficulty score with objective metrics such as operative time, complication rates and conversion rates.

## Conclusions

This study proposes the first structured expert-based classification of urological procedures performed with the SP robotic platform. The hierarchy of difficulty parallels historical laparoscopic and multi-port robotic experience, while estimated learning curves appear shorter than historical laparoscopic and multi-port benchmarks — plausibly related to cumulative expertise, a hypothesis that warrants direct testing against objective outcomes. Prior multi-port experience was considered necessary by all experts, with intensity ranging from slightly necessary to essential, before SP adoption…, and early technical challenges relate predominantly to exposure and ergonomics rather than instrument conflict. This framework may facilitate the structured dissemination of SP robotic surgery and support its safe adoption across urological centers.

## Data Availability

No datasets were generated or analysed during the current study.
